# Mechanisms underlying TDP-43 pathology and neurodegeneration: An updated Mini-Review

**DOI:** 10.3389/fnagi.2023.1142617

**Published:** 2023-03-09

**Authors:** Benjamin I. Nilaver, Henryk F. Urbanski

**Affiliations:** Division of Neuroscience, Oregon National Primate Research Center, Beaverton, OR, United States

**Keywords:** ALS, autophagy, dementia, LATE, phosphorylation, TDP-43

## Abstract

TAR DNA binding protein 43 kDa (TDP-43) plays an important role in several essential cell functions. However, TDP-43 dysfunction has been implicated in the development of various brain diseases including amyotrophic lateral sclerosis (ALS), frontotemporal lobar degeneration (FTLD), and limbic predominant age-related TDP-43 encephalopathy (LATE). Recent investigations into the individual components of TDP-43 pathology show how broader TDP-43 dysfunction may precede these disease end states, and therefore could help to explain why TDP-43 dysfunction continues to be implicated in a rapidly expanding category of neurodegenerative diseases. The literature reviewed in this article suggests that dysregulation of TDP-43 initiated by some environmental and/or genetic insults can lead to a snowballing dysfunction across the cell, involving impaired gene expression, mRNA stability, as well as the function and coordination of those pathways directly regulated by TDP-43. Furthermore, the hallmarks of TDP-43 pathology, such as hyperphosphorylation and insoluble cytoplasmic accumulation of the protein may actually be artifacts of an upstream impairment in TDP-43’s normal function. Overall, the present article summarizes current knowledge regarding TDP-43’s normal and pathological cell functions and sheds light on possible mechanisms that underlie its causal role in neurodegeneration.

## Introduction

### TDP-43 and disease

TAR DNA binding protein 43 kDa (TDP-43) is a highly conserved ubiquitously expressed nuclear protein that plays an important role in several essential cell functions, including transcriptional repression, RNA splicing, mRNA transport, microRNA maturation, translational regulation, and the formation of stress granules (Sephton et al., 2011; de Boer et al., 2020). Under normal physiological conditions, TDP-43 is almost entirely located in the nucleus but its function depends on it being shuttled between the nucleus and cytoplasm in controlled amounts (de Boer et al., 2020). Levels of TDP-43 in the cell are tightly auto regulated; i.e., TDP-43 regulates its own expression by directly binding TARDBP mRNAs (Koyama et al., 2016). Its importance to normal cell functioning is evident, as TDP-43 genomic deletion is embryonically lethal (Sephton et al., 2011).

On the other hand, pathological TDP-43 has been implicated in a wide range of neurodegenerative conditions. For example, hyperphosphorylated and ubiquitinated TDP-43 inclusions in the cytoplasm of cells in the nervous system represent a major pathological feature of amyotrophic lateral sclerosis (ALS), and frontotemporal lobar degeneration (FTLD) (Dugger and Dickson, 2017). TDP-43 pathology has also been associated with Alzheimer’s disease (AD), chronic traumatic encephalopathy (CTE), Lewy body disease (LBD), Huntington’s disease, argyrophilic grain disease (AGD), and hippocampal sclerosis (Uchino et al., 2015; de Boer et al., 2020; Eck et al., 2021). Furthermore, TDP-43 deposits have been observed in non-demented aged individuals in a condition called limbic predominant age-related TDP-43 encephalopathy (LATE) (Eck et al., 2021). In the past decade, therefore, the number of conditions associated with TDP-43 pathology has increased greatly (de Boer et al., 2020).

### TDP-43 pathology

**Figure 1:**
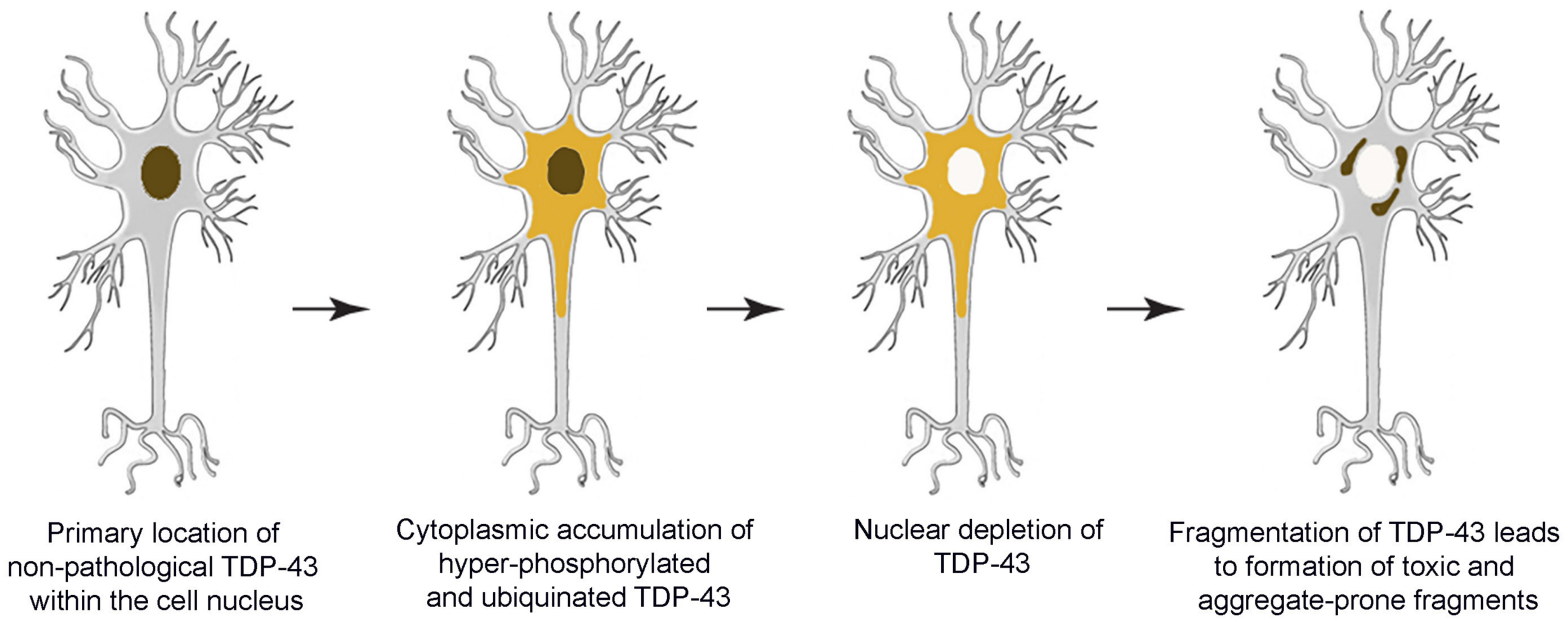
Schematic overview depicting progressive development of TDP-43 pathology within the brain.

TDP-43 pathology is usually characterized by insoluble, hyperphosphorylated and ubiquinated aggregates of TDP-43 in the cytoplasm, nucleus and cell processes of neurons and glia (Dugger and Dickson, 2017; de Boer et al., 2020). Mislocalization of TDP-43 within cellular compartments is also characteristic of the pathology (de Boer et al., 2020). Recall that normally TDP-43 is tightly auto-regulated and is almost entirely located in the nucleus. Consequently, depletion of TDP-43 in the nucleus, in association with abnormally high levels in the cytoplasm, is considered to be pathological. Indeed, TDP-43 mislocalization alone appears capable of causing mRNA instability, impaired gene regulation, mitochondrial dysfunction, impaired protein turnover, among other issues (de Boer et al., 2020). However, the underlying causes of TDP-43 mislocalization and aggregation remain unclear (Figure 1).

In the pathological condition, post-translational modifications are also typically made to the TDP-43 protein, especially hyperphosphorylation and/or fragmentation (de Boer et al., 2020). Although TDP-43 has 64 potential phosphorylation sites, and phosphorylation occurs during its normal function, pathology is associated with a degree of phosphorylation that is abnormally high (Eck et al., 2021). Common phosphorylation sites in pathological TDP-43 are Ser369, Ser379, Ser403/404, and Ser409/410, and it has been posited that these phosphorylation events have a direct causative role in pathogenesis (Eck et al., 2021). There are at least 20 disease linked missense mutations that create or remove phosphorylation sites, further implicating abnormalities in phosphorylation to pathogenesis (Eck et al., 2021).

### TDP-43 phosphorylation: Kinases and phosphatases

While hyperphosphorylated TDP-43 is associated with a variety of neurodegenerative diseases, regulated phosphorylation is necessary for TDP-43’s normal function (Eck et al., 2021). There are several kinases and phosphatases that add or remove phosphate groups from TDP-43. Currently, the kinases observed to phosphorylate TDP-43 are casein kinases 1 and 2 (CK1 and CK2), cell division cycle 7 (CDC7), and tau tubulin kinases 1 and 2 (TTBK1 and TTBK2) (Eck et al., 2021). The phosphatases that remove phosphate groups from TDP-43 are protein phosphatase 1 & 2 (PP1 and PP2), and calcinuerin (Eck et al., 2021).

Some studies aimed at ameliorating or aggravating TDP-43 pathology, by intervening with kinase and phosphatase activity, suggest that reducing TDP-43 phosphorylation can prevent accumulation and in some cases improve neurodegeneration. A study by Liachko et al. (2013) found that inhibiting the kinase CDC7 in transgenic *Caenorhabditis elegans* ALS models and human cell cultures could reduce TDP-43 phosphorylation, improve behavioral phenotypes, and reduce neurotoxicity. The authors also found that *C. elegans* with the phosphatase calcinuerin knocked out, had significantly worsened motor control phenotypes, along with phosphorylated TDP-43 aggregates, and neurodegeneration (Liachko et al., 2013). Several studies in mammalian cells treated with inhibitors of the phosphatases PP1 or calcinuerin have described an increase in phosphorylated TDP-43 aggregates (Liachko et al., 2016; Gu et al., 2018; Taylor et al., 2018). The upregulation of the kinase CK1 and the decreased activity of calcinuerin and other phosphatases has been observed in sporadic and familial ALS (Eck et al., 2021). Studies such as these have led some to posit that altering the activity of phosphatases and kinases may be a potential therapeutic intervention for ameliorating the formation of hyperphosphorylated TDP-43 deposits in TDP proteinopathies. However, evidence in support of this hypothesis is mixed and the exact roles of kinase and phosphatase enzymes in TDP-43 pathology remain to be elucidated.

### Phosphorylated TDP-43 prion-like seeding

In many diseases with pathological protein aggregates, disordered protein can act like a prion, inducing changes in other normal proteins. A study by Nonaka et al. (2016) investigated if insoluble phosphorylated TDP-43 had a prion-like seeding behavior. The authors created insoluble phosphorylated TDP-43 extracts to act as seeds, and then introduced them to neuroblastoma expressing either wild-type TDP-43 or TDP-43 lacking a nuclear localization signal (NLS)-a well characterized mutation in TDP-43 proteinopathies. The cells lacking an NLS on their own, and wild-type cells treated with the seeds were insufficient at inducing phosphorylated TDP-43 aggregates. Hyperphosphorylated aggregates could only be induced when cells lacking an NLS were exposed to seeds. Taken together, these findings suggest that it is the combination of a malfunctioning localization signaling together with exposure to insoluble phosphorylated TDP-43 aggregates that can cause a prion-like seeding behavior. These findings also provide insight into possible summation of genetic factors and spontaneous or environmental insults that may ultimately combine to play a causative role in the etiology of TDP-43 pathology.

### Phosphorylation as a compensatory response

Although insoluble hyperphosphorylated TDP-43 aggregates have been shown to be neurotoxic, this does not necessarily imply that phosphorylation itself is neurotoxic. Indeed, some studies suggest that phosphorylation of TDP-43 may actually be a compensatory response by the cell to halt or prevent TDP-43 aggregation (Brady et al., 2010; Li et al., 2011). For example, Brady et al. (2010) investigated the effect that phosphorylation of TDP-43 at site Ser409/410 in mammalian cell pathology models would have on its propensity to form insoluble aggregates. Modifying Ser409/410 on TDP-43 to a non-phosphorylatable state slightly (by 15%) reduced its tendency to aggregate. Modifying it to mimic its phosphorylated state at this site substantially (by 60%) reduced aggregation and improved TDP-43 solubility. These surprising results suggest that TDP-43 phosphorylation may actually regulate or arrest the development of insoluble TDP-43 aggregates. Brady et al. (2010) proposed that phosphorylation, particularly at Ser409/410 may happen after TDP-43 begins to aggregate, as the cell attempts to reduce further aggregation, possibly by using the electrostatic repulsion incurred by phosphorylation. Other experiments in their study showed that phosphorylation of TDP-43 was also associated with its impaired turnover. Therefore, while hyperphosphorylation at some sites may prevent aggregation by improving TDP-43’s solubility and repulsing it from other phosphorylated protein, it could also impair its degradation by protein turn over systems within the cell. A study by Li et al. (2011) reported a similar finding in mammalian cell pathology models and in transgenic *Drosophila melanogaster*. The authors found that both full-length and truncated TDP-43 had a significantly reduced propensity to aggregate when phosphomimetic substitutions were made to sites such as Ser403/404, Ser409/410, with increasing degrees of phosphorylation causing greater reduction of aggregation. They also showed that increased activity of the kinase CK2 could improve the solubility of TDP-43 fragments, a kinase that phosphorylates sites on TDP-43 associated with pathology. The studies of both Brady et al. (2010) and Li et al. (2011) suggest that hyperphosphorylation of TDP-43 at some sites associated with pathology may happen *after* the protein begins aggregating, and that it *slows* progression of the pathology. It is plausible, therefore, that hyperphosphorylation is a compensatory response by the cell to stop the aggregation of TDP-43, at the expense of poor processing by protein turnover mechanisms. So, although hyperphosphorylated TDP-43 inclusions are generally considered a primary hallmark of TDP-43 pathology, the cause and effect relationship between TDP-43 hyperphosphorylation and ultimately neurotoxicity remains unclear.

### TDP-43 dysregulation and dysfunction in pathology

There are several different components to TDP-43 pathology besides inclusions in the cytoplasm that are neurotoxic. An investigation by Yang et al. (2022) reported that low-level overexpression (less than 60% above endogenous levels) of wild-type TDP-43 in transgenic mice lead to a variety of phenotypes resembling ALS, such as neuron loss, muscle denervation, astrogliosis, oligodendrocyte injury, demyelination of the spinal cord, neuroinflammation, progressive weakness and paralysis in mid-life. Similar low-level overexpression has been observed in *post mortem* ALS and FTLD neural tissues (Yang et al., 2022). Although cytoplasmic inclusions or nuclear TDP-43 depletion was not observed in the TDP-43 overexpressing mice, the authors reported decreased solubility of TDP-43, suggesting the protein was aggregating modestly. Therefore, the findings from Yang et al. (2022) strongly suggest that pathology and ALS-like symptoms can be caused by an over expression of TDP-43, and can occur independently of the formation of large insoluble phosphorylated aggregates or nuclear depletion.

How might overexpression of TDP-43 mechanistically cause neurotoxicity, even in the absence of cytoplasmic aggregates? One possibility is that disorders of TDP-43’s normal functions could serve as a catalyst for TDP-43 pathology. Recall that TDP-43 is auto regulated; i.e., it directly binds and metabolizes TARDBP mRNAs (Wood et al., 2021). A study by Fratta et al. (2018) investigated the effect that a TDP-43 over-functioning mutation had on the transcriptome of transgenic mice. This mutation caused an increase in TDP-43’s mRNA splicing activity (i.e., a gain of function mutation (GOF)), which led to a particular set of exons being removed from direct RNA targets of TDP-43 - an event they called “skiptic exons.” These exons would normally be left in the mRNA transcripts; but due to the aberrant TDP-43 over activity they were removed. Skiptic exons caused frameshifts or premature termination codons in 30% of the RNA transcripts that were analyzed, and ultimately reduced levels of the proteins encoded by those mRNAs. The authors observed a progressive neuromuscular phenotype in the mice with the GOF mutation. However, they failed to find insoluble TDP-43 inclusions, suggesting that a GOF alone was sufficient to induce ALS like phenotypes. They also found that TARDBP mRNA levels were substantially upregulated in mice with the GOF mutation, suggesting a GOF in TDP-43’s splicing imbalances its autoregulation, although they found protein levels were not changed.

An overexpression and gain of function are not the only components to the disordered TDP-43 activity implicated in neurotoxicity. Nuclear clearance of TDP-43 is also typical of pathology, most likely stemming from decreased function in its normal roles (de Boer et al., 2020). A study by Ling et al. (2015) investigated the effect that a TDP-43 under-functioning mutation had on TDP-43’s splicing targets and overall protein expression in transgenic mice and HeLa cells. This mutation caused a decrease in TDP-43’s mRNA splicing activity, i.e., a loss of function mutation (LOF). The authors found in cells and in mice, that LOF mutation of TDP-43 resulted in mRNA transcripts containing nucleotide sequences that would normally by removed by splicing, an event called a “cryptic exon.” Cryptic exons introduced frameshifts and stop codons to the affected mRNAs.

Furthermore, Fratta et al. (2018) found that both GOF and LOF mutations induce an upregulation in TARDBP gene expression, suggesting that GOF and LOF of TDP-43 may have an effect on its autoregulation. The authors also found that the GOF and LOF mutations on TDP-43 did not act on the same set of genes. Importantly, this suggests that over functioning and under functioning in splicing by TDP-43 has its own independent effects on the transcriptome. TDP-43 GOF and LOF may play distinct roles at different stages in pathogenesis. Moreover, an increase in cryptic exon and skiptic exon splicing events has been observed in human ALS, suggesting these events play some role in full-blown human diseases (Ling et al., 2015; Fratta et al., 2018; Torres et al., 2018; Yang et al., 2022). The importance of these findings cannot be over emphasized, because up to this point the formation of phosphorylated aggregates has been a main focus of characterizing and treating TDP-43 proteinopathies. Phosphorylated aggregates might represent a late step in the pathogenesis, which is preceded by dysfunction in TDP-43 expression and normal function - and therefore suggestive of possible alternate therapeutic interventions.

### Disordered autophagy and TDP-43 aggregation

TDP-43 actively binds several thousand mRNA transcripts in its normal function and is known to modify the expression of at least 41 genes (Torres et al., 2018; Prasad et al., 2019). Gene regulation aside, how might disordered mRNA splicing by TDP-43 affect processes in the cell? Torres et al. (2018) found that down-regulating TDP-43 in human neural stem cells and HeLa cells lead to deleterious splicing events in mRNA and decreased protein levels of a critical autophagy enzyme. The authors observed a 20% increase in cryptic exons in *ATG4B* (autophagy related 4B cysteine peptidase) mRNAs, and a 30% decrease in ATG4B protein levels; changes that are consistent with a loss of TDP-43 function. Due to TDP-43’s direct binding and splicing of ATG4B mRNA, TDP-43 is considered necessary for autophagy (Torres et al., 2018). Torres et al. (2018) posited that the increase in cryptic exons likely leads to break down of aberrant mRNA’s by nonsense-mediated decay, causing the lower protein levels, and subsequent dysfunction in the autophagy response. The authors also quantified the abundance of cryptic exons in ATG4B mRNAs from ALS brain and spinal cord tissues. They found that the amount of cryptic exons in ATGB mRNAs was mainly influenced by ALS status, and that the ALS cases with higher levels of aberrant mRNAs had a more severe disease phenotype in life. TDP-43 knock out studies in mouse neuroblastoma have also reported a decreased expression of the critical autophagy gene *Atg7*, impaired autophagy, and an increase in accumulated polyubiquitinated proteins (Wood et al., 2021). In the case of over functioning, studies with mice overexpressing TDP-43 found inhibited autophagy due to an upregulation of the autophagy regulator Bcl-2 (Wood et al., 2021). Together, these findings suggest that TDP-43 over and under expression lead to a respective over or under functioning, and each on their own is sufficient to inhibit the autophagy response. TDP-43 pathology has been found in an expanding category of neurodegenerative disorders involving protein inclusions, its role in autophagy suggests a possible mechanism for TDP-43 involvement in these conditions (Wood et al., 2021).

The consequence of inhibited autophagy due to TDP-43 dysregulation may have implications for the accumulation of TDP-43 itself. For example, it has been found that TDP-43 is cleared from the cytoplasm by both the ubiquitin proteasome system (UPS) and autophagy (Wood et al., 2021). Additionally, Scotter et al. (2014) investigated the effect that inhibitors of the UPS and autophagy had on TDP-43 accumulation and solubility in human cellular ALS/FTLD models. They found that inhibition of the UPS, but not autophagy, increased levels of insoluble cytoplasmic TDP-43, as long as it began in a soluble form. Furthermore, when they inhibit both the UPS and autophagy, they found increased levels of insoluble TDP-43 macroaggregates, compared to inhibition of the UPS alone. When autophagy was inhibited, TDP macroaggregates could still be disassembled into smaller aggregate particles, although those smaller aggregates persisted in the autophagy inhibited cells, compared to controls. Taken together, Scotter et al.’s (2014) findings suggest that the UPS primarily degrades TDP-43 when it is soluble, but autophagy is required to degrade insoluble aggregate particles. Their findings suggest that inhibited autophagy, which can be caused by dysregulation in TDP-43 itself, could actually prevent the clearance and exacerbate TDP-43 aggregation (Scotter et al., 2014; Torres et al., 2018).

### Effect of TDP-43 on mRNA splicing

Other studies have identified potential disease mechanisms in TDP-43 pathology by investigating the direct transcription regulation and mRNA splicing targets of TDP-43, and how its dysfunction affects systems in the cell downstream. For example, Klim et al. (2019) investigated potential mRNA targets of TDP-43 that would be deleteriously affected by TDP-43 knock down in cultured human motor neurons and found 885 mRNA transcripts that required TDP-43 to maintain their normal levels. Of the targets they identified in human motor neurons (hMN), they noted that levels of mRNA encoding protein Stathmin-2 (STMN2) were especially sensitive to abberations in TDP-43 abundance and function; STMN2 is enriched within the central nervous system, and is essential to axonal growth and regeneration, cytoskeletal regulation, and microtubule stabilization (Klim et al., 2019). Furthermore, hMNs with TDP-43 depletion, and hMNs expressing TDP-43 with mutations commonly associated with disease, showed a significant decrease in STMN2 mRNA expression. More recently, Krus et al. (2022) generated STMN2 knockout mice and observed phenotypes that showed slow progressive, motor-selective neuropathy with functional deficits and neuromuscular denervation. Taken together, these findings emphasize that STMN2 reduction stemming from TDP-43 pathology may contribute to ALS pathogenesis by damaging the integrity of neural circuits and communication between cells, beyond impaired autophagy alone.

## Discussion

The literature investigating TDP-43’s phosphorylation, prion like seeding activity, over expression, aberrant splicing function, and role in processes like autophagy, axonal regrowth, and neurite branching paints a complex picture of TDP-43 pathogenesis. Interference at each of these components has been shown to ameliorate the accumulation of TDP-43, prevent neurodegeneration, and improve behavioral symptoms in some models. However, these studies suggest that dysfunction in TDP-43 could present itself in many ways outside of the traditional characterization of TDP-43 pathology; it is likely a mosaic of events with several discreet yet interrelated steps. The dysregulation and dysfunction of TDP-43 seems to feedback on itself and further exacerbate its own pathogenesis. Consequently, development of effective therapies for TDP-43-associated diseases may need to focus on interfering with the mechanisms that initiate this dysregulation. Eliminating only one element, for example hyperphosphorylation, may be insufficient or even harmful to disease outcomes, and could fail to overcome the adverse involvement of TDP-43 pathology in full-blown neurodegenerative diseases. These studies, taken together, suggest that the hallmarks we consider to be characteristic or even causative of TDP-43 pathology may only be the end state of a complex cascade of events with likely both environmental and genetic components. Further investigations into the factors that trigger TDP-43 dysregulation and subsequent dysfunction may elucidate more effective therapeutic targets, and enrich our understanding of the mechanisms of pathogenesis in the expanding category of diseases associated with TDP-43.

## Author contributions

BIN drafted the current manuscript, with writing and editing contributions from HFU. All authors contributed to the article and approved the submitted version.

## Funding

Funding for this article was provided by the National Institutes of Health through grants AG062220, AG066518, and OD011092.

## Conflict of interest

The authors declare that the research was conducted in the absence of any commercial or financial relationships that could be construed as a potential conflict of interest.

## Publisher’s note

All claims expressed in this article are solely those of the authors and do not necessarily represent those of their affiliated organizations, or those of the publisher, the editors and the reviewers. Any product that may be evaluated in this article, or claim that may be made by its manufacturer, is not guaranteed or endorsed by the publisher.
